# Beneficial role of oleuropein in sepsis-induced myocardial injury. Possible Involvement of GSK-3β/NF-kB pathway

**DOI:** 10.1590/ACB360107

**Published:** 2021-02-15

**Authors:** Conghui Xing, Li Xu, Yingjie Yao

**Affiliations:** 1 MS. Qingdao Municipal Hospital – Department of Cardiology – Qingdao (Shandong), China.; 2 MS. Linyi Maternal and Child Health Hospital – Department of Cardiology – Linyi (Shandong), China.

**Keywords:** Sepsis, Inflammation, Cytokines, Myocardium, Rats

## Abstract

**Purpose:**

The present study explored the potential therapeutic role of oleuropein in sepsis-induced heart injury along with the role of GSK-3β/NF-kB signaling pathway.

**Methods:**

Sepsis-induced myocardial injury was induced by cecal ligation and puncture (CLP) in rats. The cardiac injury was assessed by measuring the levels of cTnI and creatine kinase-MB (CK-MB). Sepsis-induced inflammation was assessed by measuring interleukin-6 (IL-6), IL-10 and HMGB1 levels. The different doses of oleuropein (5, 10, and 20 mg/kg) were given prior to CLP. Oleuropein (20 mg/kg) was administered after 6 hof CLP. The expressions of GSK-3β, p-GSK-3β (Ser9) and nuclear factor-κB (NF-κB) were measured in heart homogenates.

**Results:**

Cecal ligation and puncture was associated with myocardial injury, an increase in IL-6, a decrease in IL-10 and an increase in HMGB1. Moreover, it decreased the ratio of p-GSK-3β/GSK-3β and increased the expression of p-NF-kB. Pretreatment with oleuropein attenuated CLP-induced myocardial injury and systemic inflammation in a dose-dependent manner. Administration of oleuropein after the onset of CLP also attenuated cardiac injury and inflammation. It also restored CLP-induced changes in the HMGB1 levels, the ratio of p-GSK-3β/GSK-3β and expression of p- NF-kB.

**Conclusions:**

Oleuropein attenuates sepsis-induced systemic inflammation and myocardial injury by inhibiting NF-kB and GSK-3β signaling.

## Introduction

Sepsis is defined as life-threatening organ dysfunction caused by a dysregulated host response to infection^1^. It has been suggested that the development of organ dysfunction (heart, liver, kidney or lungs) may be employed as a primary outcome variable in a shock model cecal ligation and puncture in rodents^2^. Accordingly, sepsis may induce widespread damage in the different body organs including the myocardium^3^. Indeed, myocardial dysfunction is very common in patients suffering from sepsis and it is recognized as one of the major health burdens throughout the world, which may contribute to morbidity as well as mortality. The release of heart-specific biomarkers, including cardiac troponins and creatine kinase, is employed to assess sepsis-induced myocardial injury^4^^-^ 5. However, there is specific drug therapy for the management of sepsis-induced myocardial injury. Accordingly, there is a need to explore new therapeutic agents that may be potentially employment for the successful mitigation of the myocardial injury in sepsis patients.

Oleuropein is a polyphenolic compound, present in olive oil^6^, and it is found to exhibit a wide range of therapeutic activities including analgesic, anti-inflammatory, antioxidant, antidepressant, anti-Parkinson, anticancer, etc.^7^^-^ 9. Moreover, oleuropein has also been shown to produce useful effects in cardiovascular diseases including hypertension, preventing cardiomyocytes death, ischemia-reperfusion injury^10^^-^ 12. For the present investigation, it was hypothesized that the combination of cardioprotective effects and anti-inflammatory actions may possibly contribute in preventing sepsis-induced myocardial injury. However, there is no such experimental study depicting the effectiveness of oleuropein in sepsis-induced myocardial injury.

HMGB1, an intracellular protein, acts as a late mediator of inflammation and organ damage^13^^-^ 14. It exerts its proinflammatory actions via binding to receptors for advanced glycation end products (RAGE). HMGB1/RAGE axis has been implicated in several inflammatory heart disorders^15^^-^ 16. Furthermore, it has also been reported that treatment with angiotensin receptor blockers significantly inhibits the HMGB1/RAGE axis in stroke and hypertensive patients^17^. Since the molecular mechanisms of inflammatory sepsis-induced cardiac injury are not fully understood and considering the HMGB1/RAGE axis as an important target of inflammatory disorder, the present study was aimed to identify the role HMGB1/RAGE axis in sepsis-induced myocardial injury.

Glycogen synthase kinase-3β (GSK-3β) and NF-kB are the key components of intracellular signaling cascade^18^^-^ 19. The role of GSK-3β and NF-kB in the initiation and amplification of inflammation is well documented^20^^-^ 21. Their role in sepsis-induced systemic inflammation has also been described^22^^-^ 23. Furthermore, it has been suggested that oleuropein produces diverse biological actions through modulation of GSK-3β and NF-kB^24^^-^ 25. Accordingly, it has been hypothesized that oleuropein-mediated potential useful effects in sepsis-induced myocardial injury may be possibly mediated through modulation of GSK-3β and NF-kB. Accordingly, the present study was designed to explore the therapeutic potential of oleuropein in sepsis-induced myocardial injury and the possible role of GSK-3β and NF-kB in oleuropein-mediated cardioprotective effects in rats.

## Methods

### Animals and drugs

Male Wistar albino rats (200–250 g) were used for this investigation. The experiments were approved by the Ethic Committee of Qingdao Municipal Hospital with ethical approval number 2020042. The experiments were conducted as per ethical guidelines in Department of Cardiology, Qingdao Municipal Hospital, Qingdao, China. The kits for the estimation of cTnT, CK-MB, IL-6, IL-10, p-GSK-3β and p-NF-kB were purchased from MyBioSource (San Diego, CA, USA).

### Cecal ligation puncture (CLP)-induced sepsis

In this experimental sepsis model, the recommendations and considerations of the *International expert consensus for pre-clinical sepsis studies* were followed^26^. The rats were divided randomly into seven groups and sepsis was induced by CLP method as described previously^27^^-^ 28. Under anesthesia (pentobarbital sodium 20 mg/kg intraperitoneal – i.p.), the laparotomy was performed in which an incision of about a 2 cm was made to expose the cecum. Thereafter, a sterile 3-0 silk suture was used to ligate the cecum at its base. An 18-gauge needle was used to puncture the cecum and extrude fecal material from the punctured site. Afterwards, the cecum was placed back in the abdominal cavity and incision was closed using surgical sutures. Buprenorphine (0.05 mg/kg subcutaneous – s.c.) was administered to take care of surgery and sepsis-induced pain. The prewarmed normal saline (37 °C; 5 mL/100 g s.c.) was injected for fluid resuscitation. Thereafter, rats were placed in a temperature-controlled room (22°C) with 12-h light and dark cycles, with full access to water and food^29^. Twelve hours after the laparotomy, the rats were sacrificed (cervical dislocation) to remove blood (cardiac puncture method) and hearts.

### Markers of myocardial damage

Sepsis-induced myocardial injury was quantified by determining the levels of CKMB and cTnI in the plasma using commercially available diagnostic kits.

### Assessment of inflammatory markers in the circulation

The levels of IL-6 and IL10 were assessed in the plasma using commercially available ELISA kits and the estimations were done as per instructions.

*Biochemical parameters in the heart*

The heart was homogenized to quantify the levels of HMGB1, p-NF-kB, p-GSK-3 and GSK-3β using commercially available ELISA kits and the estimations were done as per instructions.

### Study design

The experimental study was comprised of seven groups, with eight rats in each group. The sample size estimation was done using “resource equation” method in which the value of E was calculated^30^. The different experimental groups included, (i) Normal; (ii) Sham, in which only laparotomy procedures were performed without cecum ligation and puncture; (iii) CLP; (iv) Oleuropein (5 mg/kg i.p.)-pretreated CLP, in which oleuropein was given 30 min before performing CLP; (v) Oleuropein (10 mg/kg i.p.)-pretreated CLP; (vi) Oleuropein (20 mg/kg i.p.)-pretreated CLP; (vii) Oleuropein (20 mg/kg)-treated CLP, in which oleuropein was administered 6 h after CLP. The dose of oleuropein in group vii was selected on the basis of results of groups iv, v and vi.

### Statistical analysis

Graph Pad Prism was employed to statistically analyze the data. The data were represented as mean ± S.D. The data were statistically analyzed using one-way ANOVA followed by Tukey’s test for *post hoc* analysis. The value of p < 0.05 was considered statistically significant.

## Results

### Cecal ligation puncture produced myocardial injury and increased systemic inflammation

**Figure 1:**
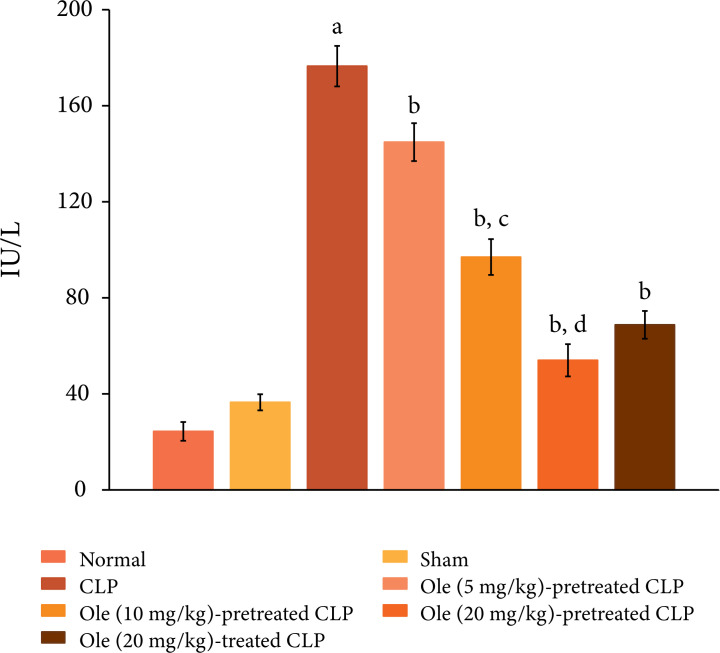
Effect of different doses of oleuropein onCLP-induced increase in plasma CK-MB levels. a = p < 0.05vs. Sham; b = p < 0.05 vs. CLP; c = p < 0.05 Ole (5 mg/kg) in CLP; d = p < 0.05 Ole (10 mg/kg) in CLP. Ole: Oleuropein.

**Figura 2:**
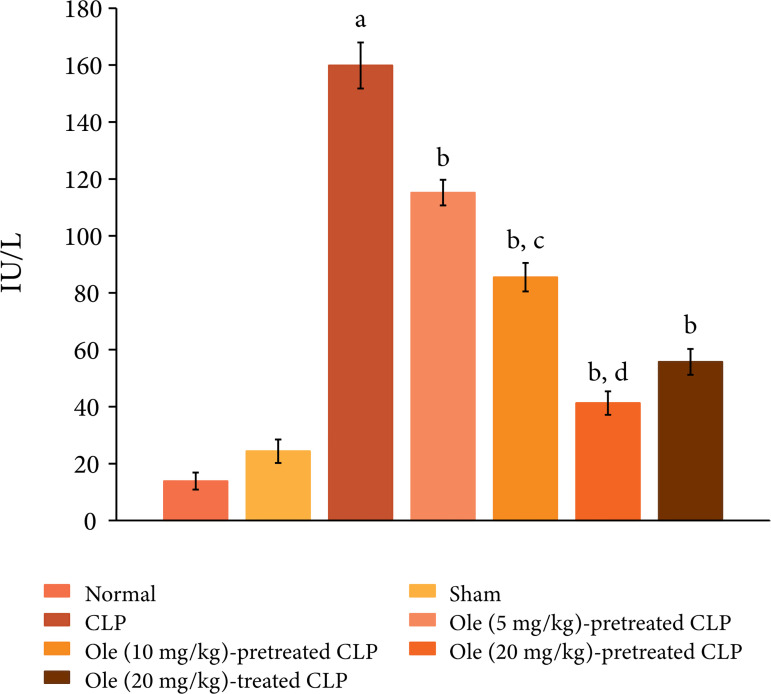
Effect of different doses of oleuropein onCLP-induced increase in plasma cTnT levels. a = p < 0.05 vs. Sham; b = p < 0.05 vs. CLP; c = p < 0.05 Ole (5 mg/kg) in CLP; d = p < 0.05 Ole (10 mg/kg) in CLP.

**Figure 3:**
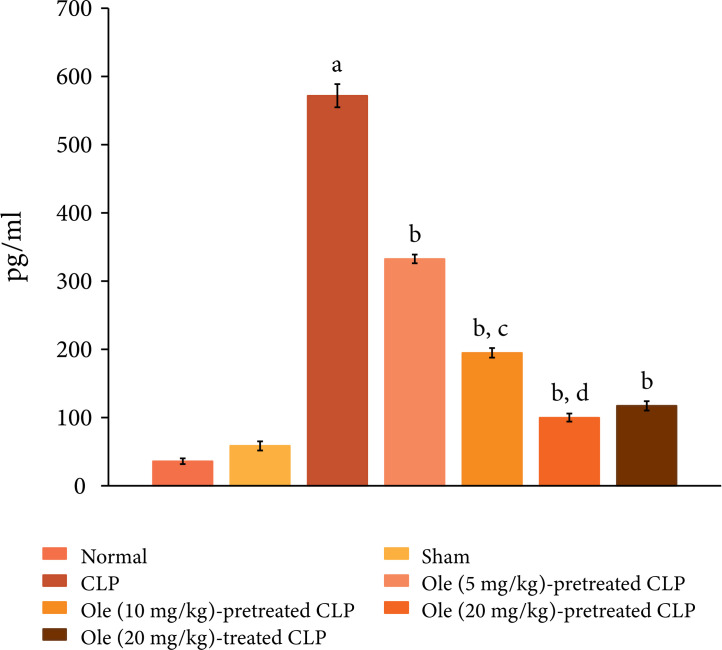
Effect of different doses of oleuropein on CLP-induced increase in plasma IL-6 levels. a = p < 0.05 vs. Sham; b = p < 0.05 vs. CLP; c = p < 0.05 Ole (5 mg/kg) in CLP; d = p < 0.05 Ole (10 mg/kg) in CLP.

**Figure 4:**
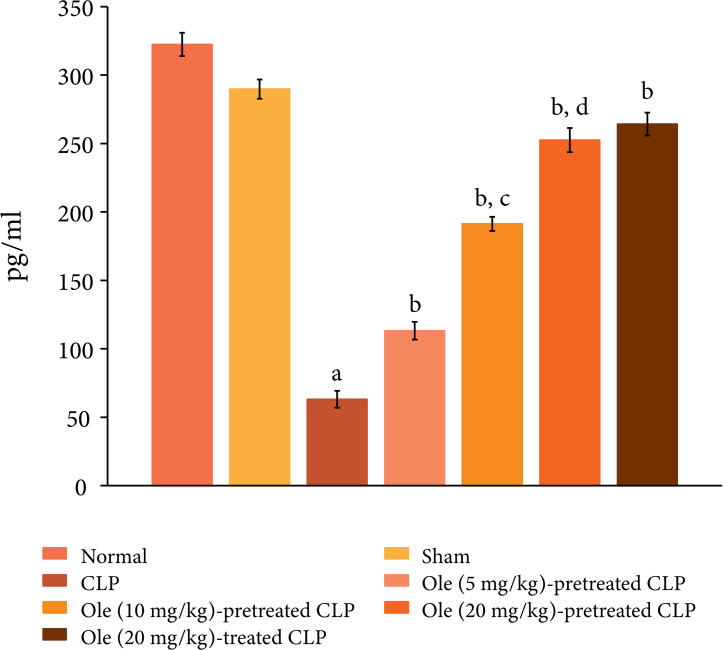
Effect of different doses of oleuropein on CLP-induced increase in plasma IL-10 levels. a = p < 0.05 vs. Sham; b = p < 0.05 vs. CLP; c = p < 0.05 Ole (5 mg/kg) in CLP; d = p < 0.05 Ole (10 mg/kg) in CLP.

The ligation and puncture of the cecum led to marked myocardial injury, which was assessed 12 h after laparotomy. Indeed, there was a significant increase in the plasma levels of CK-MB (Fig. 1) and cTnT (Fig. 2) as compared to sham and normal control. Creatine kinase-MB and cTnT are the heart-specific biochemicals and their increase in the plasma levels indicates injury to the heart. Moreover, CLP also induced systemic inflammation and there was a significant increase in the plasma levels of proinflammatory cytokine, IL-6 (Fig. 3) and decrease in the plasma levels of anti-inflammatory cytokine, IL-10 (Fig. 4).

### Oleuropein attenuated CLP-induced myocardial injury and systemic inflammation

Pretreatment with oleuropein (5, 10 and 20 mg/kg) abolished CLP-induced increase in the plasma levels of CK-MB (Fig. 1) and cTnT (Fig. 2) in a dose-dependent manner suggesting the cardioprotective actions of oleuropein. Moreover, pretreatment with oleuropein was also associated with a significant decrease in the plasma levels of IL-6 (Fig. 3) and increase in the IL-10 levels (Fig. 4) in CLP-subjected rats suggesting the anti-inflammatory actions of oleuropein. Administration of oleuropein (20 mg/kg) after the induction of CLP was attenuated myocardial injury (Figs. 1 and 2) and inflammation. There was significant decrease in the levels of IL-6 (Fig. 3) and rise in the levels of IL-10 (Fig. 4) in the plasma of oleuropein-treated rats.

### Oleuropein abolished CLP-induced biochemical changes in the heart

**Figure 5:**
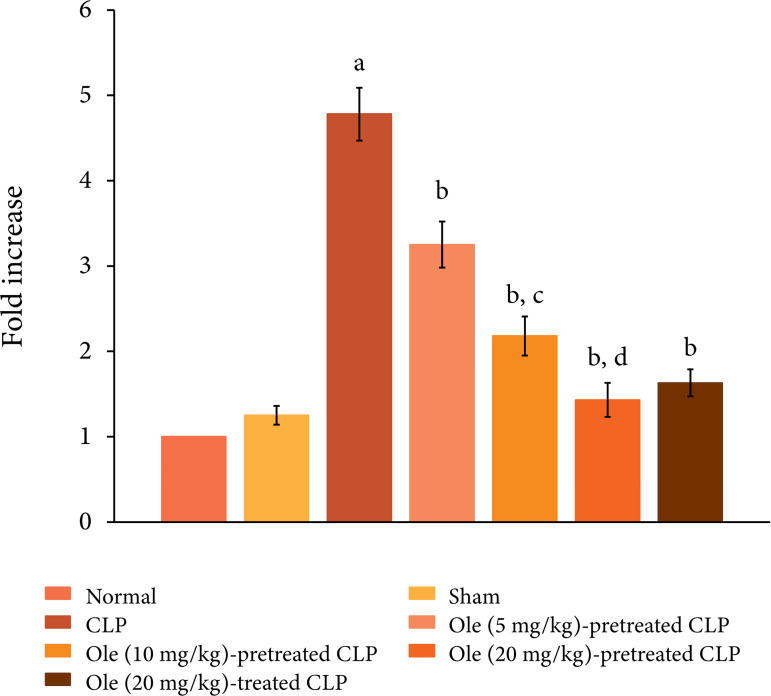
Effect of different doses of oleuropein on CLP-induced increase in HMGB1 levels in the heart homogenate. a =p < 0.05 vs. Sham; b = p < 0.05 vs. CLP;c = p < 0.05 Ole (5 mg/kg) in CLP; d = p < 0.05 Ole(10 mg/kg) in CLP.

**Figure 6:**
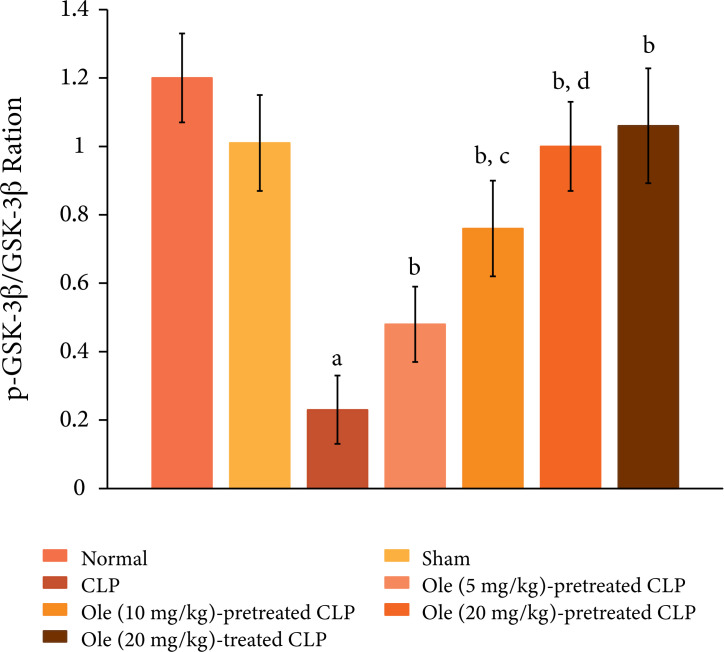
Effect of different doses of oleuropein onCLP-induced changes in p-GSK-3β/GSK-3β ratio in the heart homogenate. a = p < 0.05 vs. Sham; b = p < 0.05 vs. CLP; c = p < 0.05 Ole (5 mg/kg) in CLP; d = p < 0.05Ole (10 mg/kg) in CLP.

**Figure 7:**
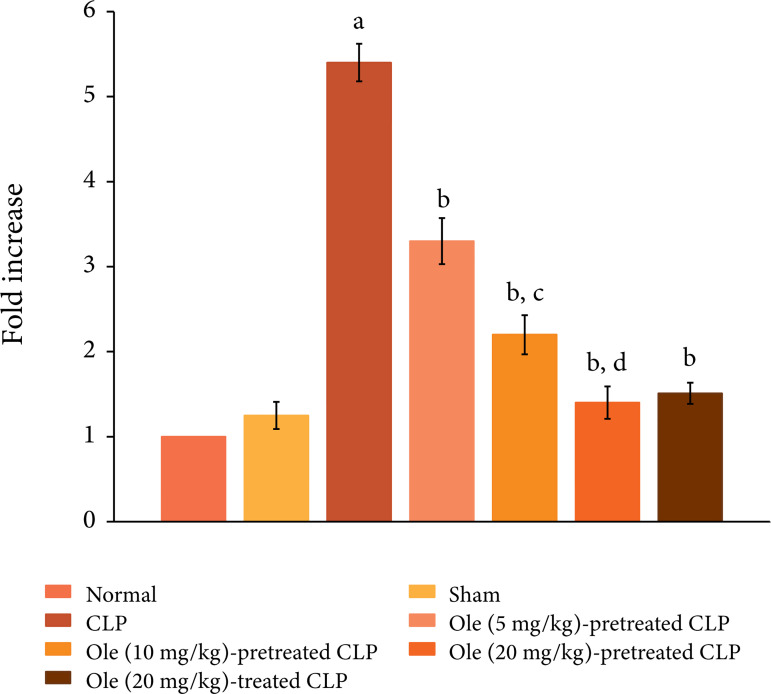
Effect of different doses of oleuropein onCLP-induced changes in p-Akt levels in the heart homogenate. a = p < 0.05 vs. Sham; b = p < 0.05 vs. CLP; c = p < 0.05 Ole (5 mg/kg) in CLP; d = p < 0.05 Ole(10 mg/kg) in CLP.

The ligation and puncture of cecum produced significant changes in the heart homogenates including an increase in protein expression of HMGB1 (Fig. 5), a decrease in the ratio of p-GSK-3β/GSK-3β (Fig. 6) and increase in the expression of p-NF-kB (Fig. 7). HMGB1 is a cytokine and is considered as a specific marker of sepsis. The decrease in the levels of p-GSK-3β indicates the activation of enzyme GSK-3β, while the increase in the levels of p-NF-kB indicates its increase inactivity. Pretreatment with oleuropein (5, 10 and 20 mg/kg) led to the restoration of CLP-induced changes in the HMGB1 levels, the ratio of p-GSK-3β/GSK-3β and expression of p-NF-kB in a dose-dependent manner. Administration of oleuropein (20 mg/kg) following the induction of CLP also led to restoration of biochemical alterations including HMGB1 levels (Fig. 5), p-GSK-3β/GSK-3β ratio and p-NF-kB levels (Fig. 6).

## Discussion

In this current study, ligation and puncture of cecum produced marked myocardial injury, which was assessed 12 h after laparotomy by measuring the heart-specific biochemicals, i.e., CK-MB and cTnT in the circulation. Cecal ligation and puncture-induced myocardial injury may be ascribed to the development of sepsis as sepsis is one of the important factors contributing to myocardial injury^31^. Moreover, CLP is one of the most common methods to induce sepsis in laboratory animals^28^. Accordingly, it is possible to suggest that CLP-induced development of sepsis may be responsible for the development of myocardial injury. Cecal ligation and puncture-induced myocardial injury, observed in this study, is consistent with the earlier published reports^32^^-^ 33. In this study, CLP also led to the development of systemic inflammation, which was assessed by decreased levels of IL-10 (anti-inflammatory cytokine) and an increase in IL-6 (proinflammatory cytokine) in the circulation. Along with it, there was a significant rise in the levels of HGMB in the heart homogenates following CLP. HMGB1 is one of the specific markers of sepsis and it has been suggested to contribute in myocardial injury. Indeed, HMGB is a late mediator of sepsis and its release has been detected after 8 h of sepsis^34^. There have been previous studies showing that CLP leads to development of systemic inflammation, which may contribute to myocardial injury^35^.

In this investigation, pretreatment with different doses of oleuropein led to significant amelioration of CLP-induced increase in the plasma levels of CK-MB and cTnT, suggesting its cardioprotective actions. Moreover, pretreatment with oleuropein also ameliorated CLP-induced increase in IL-6 and a decline in IL-10 levels in the plasma, suggesting the decline in systemic inflammation. Apart from it, oleuropein also led to a significant decline in the levels of HMGB1 in the heart homogenates suggesting its ameliorative effects on sepsis. Apart from it, administration of oleuropein after the induction of CLP (as post-treatment) also produced cardioprotection and attenuated the levels ofHMGB1. There have been previous studies documenting that oleuropein exerts anti-inflammatory actions and it serves to reduce the levels of proinflammatory cytokines in circulation^36^^-^ 37. Based on this, it may be possibly suggested that an oleuropein-mediated decrease in systemic inflammation may contribute in attenuating myocardial injury in CLP-subjected rats. There have been studies showing the widespread use of oleuropein in experimental diseases such as in pain, periodontitis, asthma, renal injury^36^^-^ 38. Moreover, oleuropein is also documented to exert a protective effect on the heart as it is found to prevent heart failure, ischemia-reperfusion myocardial injury and arrhythmia^39^^-^ 41. Nevertheless, the beneficial actions of oleuropein in sepsis-induced myocardial injury are reported for the first time in this current study.

In this investigation, CLP also led to an increase in the expression of p-NF-kB and a decrease in the ratio of p-GSK-3β/GSK-3β. NF-kB is a key transcriptional factor and its activation, as assessed by an increase in expression of its phosphorylated form, has been identified in sepsis, including in CLP model^42^. Activation of NF-kB plays an important role in the initiation and maintenance of inflammatory reactions^43^. Moreover, an increase in p-NF-kB and its subsequent activation has been associated with the development of myocardial injury^44^. Accordingly, it is possible that CLP-induced activation of NF-kB signaling may play a key role in initiating inflammation and producing myocardial injury. However, pretreatment as well as post-treatment of oleuropein led to the significant restoration of p-NF-kB in the heart homogenates of CLP-subjected rats. Oleuropein has been shown to attenuate the production of inflammatory cytokines by inhibiting the activation of NF-kB^45^. Accordingly, it may be proposed that oleuropein-mediated decrease in NF-kB signaling may contribute in reducing inflammation and producing myocardial protection.

GSK-3β is a unique enzyme which gets deactivated after its phosphorylation; hence, the decrease in the ratio of p-GSK-3β/GSK-3β represents the increase in the activation of GSK-3β^46^. Accordingly, in the current investigation, the decrease in the ratio of p-GSK-3β/GSK-3β represents the increase in the GSK-3β enzymatic activity in CLP-subjected rats. The activation of GSK-3β has been postulated in CLP-induced sepsis and liver injury^47^. However, it is the first report documenting the activation of GSK-3β in sepsis-induced inflammation and heart injury. Treatment with oleuropein abolished CLP-induced decrease in p-GSK-3β/GSK-3β ratio suggesting the inactivation of GSK-3β in response to oleuropein treatment. A preclinical study has described that oleuropein offers neuroprotective effects by inhibiting the GSK-β activity^33^. Accordingly, it may be proposed that oleuropein may prevent sepsis-induced cardiac injury by inhibiting systemic circulation, which may be possibly attributed to a decrease in NF-kB and GSK-3β signaling. However, future studies are required to elucidate the precise pathway involved in oleuropein-mediated beneficial effects in CLP-subjected rats. Since sepsis leads to multi-organ dysfunction, therefore, the protective role of oleuropein on different organs including brain, liver and lungs may also be investigated in CLP model in future studies.

## Conclusions

Oleuropein has the potential to prevent and treat sepsis-induced systemic inflammation and myocardial injury. The protective effects of oleuropein in sepsis-subjected rats may be attributed to inhibition of NF-kB and GSK-3β signaling.
